# The Role of Esaxerenone in the Continuum of Heart Failure With Preserved Ejection Fraction: Insights From a Prospective Observational Study

**DOI:** 10.1002/clc.70137

**Published:** 2025-04-24

**Authors:** Takashi Naruke, Miho Hashimura‐Kakogawa, Yoichi Suzuki, Yuki Ikeda, Lisa Kitasato, Takeru Nabeta, Yuki Usami‐Naruke, Shunsuke Ishii, Jun Oikawa, Ryo Kameda, Yoshiyasu Minami, Hidehira Fukaya, Masaru Yuge, Takeo Kawaguchi, Junya Ako

**Affiliations:** ^1^ Department of Cardiovascular Medicine Kitasato University School of Medicine Sagamihara Kanagawa Japan; ^2^ Department of Cardiovascular Medicine Odawara Municipal Hospital Odawara Kanagawa Japan; ^3^ Department of Diabetology and Metabolism Fujisawa Shonandai Hospital Fujisawa Kanagawa Japan; ^4^ Aya Clinic Japan; ^5^ Department of Kitasato Clinical Research Center Kitasato University School of Medicine Sagamihara Kanagawa Japan

**Keywords:** aortic stiffness, diastolic dysfunction, Esaxerenone, HFpEF, non‐steroidal mineralocorticoid receptor antagonist, NT‐proBNP, pre‐HFpEF

## Abstract

**Background:**

Heart failure with preserved ejection fraction (HFpEF) presents significant therapeutic challenges, particularly exacerbated by comorbidities such as hypertension and diabetes. The modulation of the Renin–Angiotensin–Aldosterone System is critical in managing HFpEF progression. Esaxerenone (ESAX), a selective mineralocorticoid receptor antagonist, may offer benefits in managing HFpEF continuum due to its unique mechanism.

**Methods:**

Conducted at Odawara Municipal Hospital, this single‐center, prospective, observational study involved hypertensive adult outpatients diagnosed with either type 2 diabetes mellitus or chronic kidney disease. Patients were categorized into HFpEF and pre‐HFpEF groups based on established criteria. The study primarily assessed changes in blood pressure and cardiac function (through NT‐proBNP levels and echocardiography), along with secondary outcomes including aortic stiffness and oxidative stress over a 24‐week period.

**Results:**

Both HFpEF and pre‐HFpEF patients exhibited significant reductions in blood pressure, with no significant differences between the patients. HFpEF patients experienced decreases in NT‐proBNP levels and oxidative stress similar to those in pre‐HFpEF patients. Notably, pre‐HFpEF patients demonstrated more pronounced improvements in cardiac function, particularly in the E/e' ratio and global longitudinal strain, compared to HFpEF patients. Additionally, 30% of pre‐HFpEF patients had improved to stage A, suggesting potential for early intervention.

**Conclusions:**

ESAX appears to be effective in managing the heart failure continuum, particularly benefiting pre‐HFpEF patients. Its impacts suggest the potential benefits of early intervention in decelerating disease progression and potentially preventing the new onset of HFpEF, highlighting the importance of targeted therapies at early stages of heart failure.

## Graphical abstract

Study Overview: Prospective study on Esaxerenone in HFpEF with 67 patients showing improvements in blood pressure, aortic stiffness, and especially cardiac function in pre‐stage patients. Safety was comparable to prior reports. Conclusion: Esaxerenone is effective in HFpEF treatment, particularly promising for early intervention.

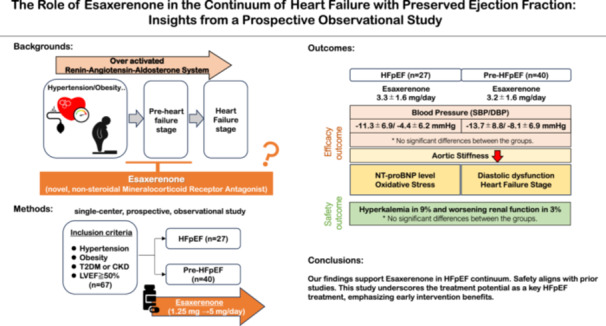

## 1 Introduction

Heart failure, particularly heart failure with preserved ejection fraction (HFpEF), has become a growing global public health concern [1]. HFpEF, characterized by preserved left ventricular ejection fraction (LVEF), is associated with multiple comorbidities, including hypertension (HTN), obesity, and diabetes mellitus [2]. Until recently, there have been no approved or effective therapies proven to reduce mortality. However, considering the documented heart failure continuum precipitated by these comorbidities [3], initiating interventions at an early stage of heart failure is deemed appropriate and potentially beneficial [4]. Moreover, the coexistence of these comorbidities leads to the overactivation of the Renin–Angiotensin–Aldosterone System (RAAS), which is associated with the progression of heart failure [3, 4]. It is therefore not surprising that modern cardiovascular medicine regards the control of the RAAS as the cornerstone of intervention [4, 5, 6]. Notably, mineralocorticoid receptors (MR) expressed in cardiomyocytes and vascular endothelial cells play a key role in the RAAS activity and are closely linked to the pathogenesis of the HFpEF and its progression [4, 5]. Previously, the steroidal and nonselective MR antagonist (MRA), spironolactone has demonstrated beneficial effects in patients with hypertension and HFpEF via RAAS or MR modulation [7]. However, its use is associated with an increased risk of hyperkalemia, which can contribute to cardiovascular and all‐cause mortality [8]. A nonsteroidal and selective MRA, Esaxerenone (ESAX), has been developed to overcome the limitations of conventional MRAs, offering promising antihypertensive effects [9, 10]. Therefore, we aim to provide further data on the therapeutic advantages and safety of ESAX, emphasizing its potential role in the HFpEF continuum.

## 2 Methods

### 2.1 Study Design

This single‐center, prospective, observational study was conducted at the Odawara Municipal Hospital from January 2021 to September 2023. Its primary aim was to evaluate the efficacy and safety of ESAX in managing the HFpEF continuum. The study was approved by the Ethics Committee of Odawara Municipal Hospital and conducted in accordance with the Declaration of Helsinki. Informed consent was obtained from all participants before their enrollment in the study.

### 2.2 Study Population

Adult outpatients aged 18 years and older were recruited based on the following three inclusion criteria.

Hypertension classification: Patients with grade 1 or 2 hypertension, exhibiting office systolic blood pressure (SBP) between 140 and 179 mmHg and/or diastolic blood pressure (DBP) 90–109 mmHg, and home SBP of 135–159 mmHg and/or DBP of 85–99 mmHg.

Body mass index (BMI): Patients with a BMI over 25 kg/m².

Comorbidities: Patients diagnosed with type 2 diabetes mellitus (T2DM) with hemoglobin A1c (HbA1c) levels between 7.0% and 10.0% in the 6 months before recruitment despite drug treatment, or chronic kidney disease (CKD) characterized by estimated glomerular filtration rate (eGFR) below 60 mL/min/1.73 m² and/or urine albumin–creatinine ratio (UACR) above 30 mg/g Cr for over 3 months [11]. Among them, patients with LVEF ≥ 50% were categorized into HFpEF or pre‐HFpEF.

In this study, HFpEF patients were identified as those meeting the criteria of NYHA class > II and the following established criteria for HFpEF, as previously reported: (A) NT‐proBNP > 220 pg/mL (sinus rhythm) or > 660 pg/mL [atrial fibrillation (AF)]; and (B) left atrium volume index (LAVI) > 34 mL/m^2^ or left ventricular mass index (LVMI) ≥ 149 g/m^2^ in men or ≥ 122 g/m^2^ in women; and (C) septal e′ wave velocity < 7 cm/s or average E/e′ ratio ≥ 15 [12]. Conversely, patients were classified as pre‐HFpEF if they failed to meet any one of the above criteria.

We defined AF, including paroxysmal AF, in accordance with the most recent guidelines [13, 14]. Exclusion criteria included orthostatic hypotension, cardiovascular disease or intervention within the previous 6 months, serum potassium (K^+^) level < 3.5 mEq/L or ≥ 5.1 mEq/L, eGFR < 30 mL/min/1.73 m^2^, nephrotic syndrome, and significant valvular heart disease as any valvular abnormality requiring surgical or percutaneous intervention or causing hemodynamic compromise as evidenced by abnormal echocardiographic parameters.

### 2.3 Treatment and Follow‐Up

ESAX was administered once daily in the morning in all patients. The initial dose was 1.25 mg and titrated to 5 mg if required to achieve blood pressure targets according to current Japanese Society of Hypertension Guidelines. Specifically, we aimed for a home BP of < 125/75 mmHg and an office BP of < 130/80 mmHg [15]. The study did not permit any changes in the type or dosage of concomitant antihypertensive medications during the study period. In cases treated with MRAs before ESAX administration, ESAX was administered at least 4 weeks after the cessation of MRAs. All patients were followed through clinic visits at least every 2, 4, 8, 12, and 24 weeks. When patients complained of symptoms such as fainting or dizziness, the dose of ESAX was maintained, reduced in half, or terminated at a clinician's discretion. If any contraindications listed in the drug package insert occurred, ESAX was immediately discontinued.

### 2.4 Outcomes

The primary efficacy endpoints were the changes in blood pressure levels and improvements in cardiac function, assessed through NT‐proBNP values and echocardiographic findings, from baseline to 24 weeks. The secondary endpoints included changes in aortic stiffness, evaluated using bilateral brachial‐ankle pulse wave velocity (baPWV), and oxidative stress, assessed by d‐ROMs, from baseline to 24 weeks. Safety outcomes included the incidence of adverse effects such as severe hypotension, significant hyperkalemia, and worsening renal function.

### 2.5 Detailed Methodological Approaches

#### 2.5.1 Blood Pressure Measurements and Laboratory Tests

The measurement of home BP twice daily within 1 h after waking was encouraged. Each measurement was conducted after urination, before taking morning medications, and after 1–2 min of seated rest [15], then the average daily BPs were calculated. The home SBP and DBP were defined as the average of the latest 2 weeks of each visit of the daily home SBP and DBP. Clinical laboratory tests were performed at baseline and at 2, 4, 8, 12, and 24 weeks in all patients. NT‐proBNP was measured at baseline and at 4, 8, 12, and 24 weeks while UACR was measured at baseline and subsequently at 12 and 24 weeks.

#### 2.5.2 Echocardiography

Transthoracic echocardiography was performed at baseline and 24 weeks using an EPIQ 7G ultrasound system with X5‐1 matrix array transducer (Philips Healthcare, Philips Medical Systems, Andover, MA, USA). These assessments were performed by two experienced physicians who were blinded to the participants' clinical data, achieving an interobserver concordance greater than 95%. This ensured unbiased interpretations of the echocardiographic data.

Using conventional echocardiography, left ventricular (LV) dimensions and LVEF were measured. These assessments included peak velocities of transmitral early (E) and late diastolic (A) LV filling, and the E/A ratio. The measurements of LV dimensions, left ventricular mass (LVM), and LVEF adhered to the joint guidelines of the American Society of Echocardiography [16]. The LVMI was calculated by indexing LVM to body surface area. LAVI was determined using the biplane method of disks from standard apical 2‐ and 4‐chamber views at end‐systole, ensuring that pulmonary vein ostia and the left atrial appendage were excluded. All volumes were indexed to the body surface area, expressed in m² [16, 17]. For diastolic function assessment, pulsed Doppler mitral inflow velocities were obtained by positioning a 1–2 mm sample volume between the mitral leaflet tips in the apical four‐chamber view, with the Doppler beam aligned parallel to the blood flow. Tissue Doppler Imaging (TDI) was performed at the septal mitral annulus, capturing early (e′) diastolic velocities. The E/e′ ratio, representing the transmitral to mitral annular early diastolic velocity, was also calculated. Global longitudinal strain (GLS) was measured using QLab 13.0 software's automatic speckle‐tracking capabilities. Each measurement was visually verified and manually adjusted if necessary to ensure precision. Poor quality datasets were excluded from the analysis. GLS values were averaged from apical four‐, three‐, and two‐chamber views.

Morphological assessments in this study included LAVI and LVMI measurements, while functional parameters evaluated were septal e′ wave velocity, E/e′ ratio, and GLS. These parameters provided comprehensive insights into cardiac structure and function, establishing thresholds for cardiac structural and functional abnormalities based on recent guideline. Specifically, LAVI ≥ 29 mL/m² for morphology, LVMI > 116 (men)/95 (women) g/m² for structural changes, GLS < 16%, an average E/eʹ ≥ 15, or septal e′ velocity < 7 cm/s for ventricular function [18].

#### 2.5.3 Aortic Stiffness

The baPWV was measured on baseline and 24 week using a measurement device (BP‐203RPE II; Omron Colin, Tokyo, Japan). The highest baPWV value from the bilateral measurements was used in the analysis. The normal reference value is less than 1400 cm/s [19].

#### 2.5.4 Oxidative Stress Measurement

The d‐ROMs test was performed at baseline and at 24 weeks using the Free Radical Analytical System (Diacron, Grosseto, Italy), as reported previously [20]. Briefly, in a pipette, 25 µL of the serum sample was mixed with an acetic acid buffered solution (pH 4.8) to fix the hydrogen ion concentration; a chromogenic substrate was then added to the mixture. In an acidified medium, both bivalent and trivalent iron from the blood protein ionize and work as catalysts to break down the hydroperoxide component in the serum into alkoxyl and peroxy radicals to become free radicals. Next, the mixture was incubated in the thermostatic block of the system, followed by its transfer into a cuvette, including colorless chromogen. Subsequently, the chromogen was oxidized by free radicals to a radical cation, which was magenta in color. The intensity of the magenta color reflects the concentration of hydroperoxide in the serum; this was measured using a photometer after centrifuging the prepared sample for 1 min. The measured value was expressed as U.CARR (1 U.CARR = 0.08 mg/dL H_2_O_2_). The normal reference level of d‐ROMs is 250 to 300 U.CARR [20].

#### 2.5.5 Safety Monitoring

The safety endpoints assessed the frequency of adverse events, including symptomatic dizziness, attainment of systolic blood pressure < 90 mmHg, hyperkalemia (defined as serum K^+^ ≥ 5.5 mEq/L), severe hyperkalemia (defined as serum K^+^ ≥ 6.0 mEq/L or ≥ 5.5 mEq/L in two consecutive measurements) [21, 22], the incidence of worsening renal function (WRF) (defined as reaching eGFR < 30 mL/min/m^2^), and overall tolerability of ESAX.

#### 2.5.6 Statistical Analysis

The analysis set included patients who received ≥ 1 dose of ESAX and had at least one efficacy outcome measurement. The analysis of the discontinuation cases was conducted only with the latest available clinical data until the visit just before the discontinuation. Continuous variables were compared using the Mann–Whitney *U* test for non‐normal distributed data and the *t*‐test for normal distributed data. Paired *t*‐tests were employed to compare sequential values between the baseline and follow‐up measurements. The data were presented as medians with interquartile ranges or means ± standard deviations as appropriate. Dichotomous variables were compared using the chi‐squared or Fisher's exact test and were presented as percentages. All statistical analyses were performed using JMP 11.1.1 (SAS Institute, Cary, NC, USA). All *p*‐values were two‐sided, and *p* < 0.05 was considered statistically significant.

## 3 Results

### 3.1 Patient's Backgrounds

**FIGURE 1:**
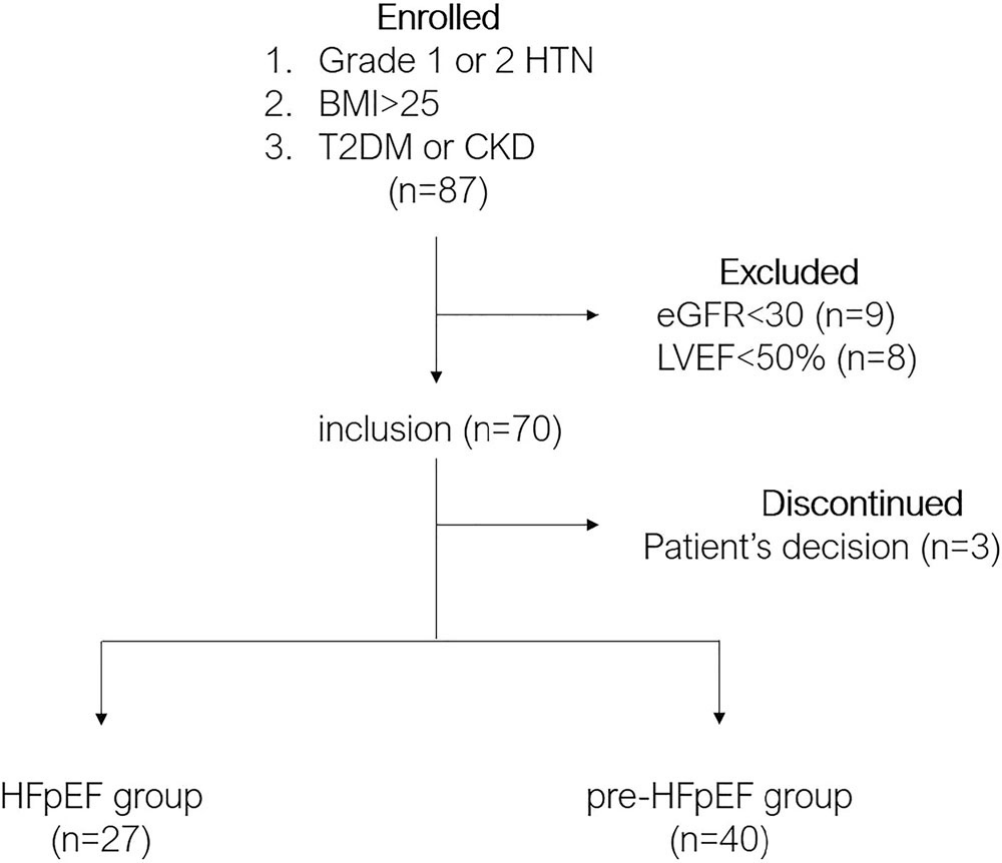
Study design. Grade 1 or 2 HTN indicates grade 1 or 2 hypertension; BMI, body mass index; CKD, chronic kidney disease; eGFR, estimated glomerular filtration rate; LVEF, left ventricular ejection fraction; T2DM, type 2 diabetes mellitus.

Among 87 eligible patients, we finally included 67 patients in the present study (Figure 1). Patient characteristics are described in Table 1. The majority of patients were male (87%), and the mean age was 63.7 years old. The mean home SBP and DBP at baseline were 138.3 ± 7.7 and 83.8 ± 6.5 mmHg. The prevalence of T2DM complications was about 60%, with an average duration of 4.5 years and an average HbA1c level of 6.8% ± 0.7%. The prevalence of CKD was about 60%, and the mean eGFR was 61.0 ± 14.7 mL/min/1.73 m^2^. The serum K^+^ level was 4.2 ± 0.2 mEq/L. Baseline cardiovascular medications included loop diuretics, RAAS inhibitors, sodium‐glucose co‐transporter‐2 (SGLT2) inhibitors, and statin in 43%, 100%, 52%, and 87%, respectively.

**TABLE 1:** Baseline characteristics.

	Total (*n* = 67)	HFpEF (*n* = 27)	Pre‐HFpEF (*n* = 40)	*p* value
Demographic characteristics				
Male, *n* (%)	58 (87%)	24 (89%)	34 (85%)	0.723
Age (years old)	63.7 ± 9.8	67.3 ± 7.0	61.2 ± 10.7	0.011
Physiological measurements				
Home SBP (mmHg)	138.3 ± 7.7	140.0 ± 8.8	137.1 ± 6.6	0.123
Home DBP (mmHg)	83.8 ± 6.5	84.1 ± 8.3	83.6 ± 5.0	0.753
BMI (kg/m^2^)	29.2 ± 3.5	28.7 ± 3.7	29.5 ± 3.4	0.348
Past medical history				
Atrial fibrillation, *n* (%)	16 (24)	13 (48)	3 (8)	< 0.001
Ischemic heart disease, *n* (%)	25 (37)	12 (44)	13 (33)	0.440
Diabetes mellitus, *n* (%)	42 (63)	17 (63)	25 (63)	1.000
Duration of diabetes, year	4.5 ± 2.1	4.5 ± 1.4	4.5 ± 2.1	0.891
Chronic Kidney Disease, *n* (%)	43 (64)	22 (81)	21 (53)	0.020
Diabetic Kidney Disease, *n* (%)	21 (31)	12 (44)	9 (23)	0.07
Laboratory findings				
eGFR (mL/min/1.73 m^2^)	61.0 ± 14.7	56.2 ± 15.4	64.1 ± 13.6	0.029
Serum potassium (mEq/L)	4.2 ± 0.2	4.3 ± 0.2	4.2 ± 0.2	0.513
HbA1C (%)	6.8 ± 0.7	6.5 ± 0.6	7.0 ± 0.7	0.002
NT‐proBNP (pg/mL)	449.6 ± 679.3	877.6 ± 873.0	129.6 ± 61.8	< 0.001
Medication and other intervention				
Loop diuretics, *n* (%)	29 (43)	19 (70)	10 (25)	< 0.001
RAAS inhibitor, *n* (%)	67 (100)	27 (100)	40 (100)	1.000
ACEi, *n* (%)	7 (10)	2 (7)	5 (13)	0.201
ARB, *n* (%)	23 (34)	8 (30)	15 (38)	0.603
ARNI, *n* (%)	37 (55)	17 (63)	20 (50)	0.327
Switch from MRAs, *n* (%)	23 (34)	9 (33)	14 (35)	1.000
CCB, *n* (%)	24 (36)	10 (37)	14 (35)	1.000
SGLT2 inhibitors, *n* (%)	35 (52)	15 (56)	20 (50)	0.803
Stain, *n* (%)	58 (87)	25 (93)	33 (83)	0.295
Glucose‐lowering therapy				
Insulin, *n* (%)	6(9)	2 (7)	4 (10)	1.000
GLP‐1RAs, *n* (%)	7 (10)	4 (15)	3 (8)	0.427
Echocardiography				
LVEF (%)	58.5 ± 6.1	57.6 ± 6.7	59.2 ± 10.6	0.257
LAVI (mL/m^2^)	40.7 ± 9.2	45.6 ± 10.5	36.3 ± 7.5	< 0.001
LVMI (g/m^2^)	102.2 ± 29.9	111.5 ± 37.0	92.3 ± 21.5	0.006
e′ wave velocity (cm/s)	6.1 ± 1.6	5.2 ± 1.2	7.1 ± 1.8	< 0.001
E/e' ratio	12.5 ± 3.5	14.0 ± 3.9	11.1 ± 2.8	< 0.001
TRPG (mmHg)	21.7 ± 9.4	25.1 ± 12.0	18.5 ± 5.0	0.001
GLS (%)	15.3 ± 2.6	14.5 ± 2.1	16.2 ± 3.8	0.006
Aortic stiffness				
baPWV (cm/s)	1677.7 ± 368.1	1656.3 ± 338.8	1691.6 ± 410.3	0.709
Oxidative stress marker				
d‐ROMs	324.6 ± 57.5	360.5 ± 51.3	288.6 ± 38.3	< 0.001

*Note:* Data are mean ± standard deviation.

Abbreviations: ACEi, angiotensin‐converting enzyme inhibitors; ARB, angiotensin receptor blockers; ARNI, angiotensin receptor‐neprilysin inhibitor; baPWV, Bilateral brachial‐ankle pulse wave velocity; BMI, body mass index; CCB, calcium channel blocker; DBP, diastolic blood pressure; eGFR, estimated glomerular filtration rate; GLP‐1 RAs, GLP‐1 receptor agonists; GLS, global longitudinal strain; HbA1c, hemoglobin A1c; LAVI, left atrial volume index; LVEF, left ventricular ejection fraction; LVMI, left ventricular mass index; MRAs, mineralocorticoid receptor antagonists; RAAS, renin–angiotensin–aldosterone system; SBP indicates systolic blood pressure; SGLT2, sodium‐glucose co‐transporter‐2; TRPG, tricuspid regurgitation peak gradient; UACR, urine albumin–creatinine ratio.

Among the study cohort, we identified that HFpEF patients were 27 patients (40%). These patients were older, had higher frequency of AF, worse renal function, and higher NT‐proBNP levels than those in the pre‐HFpEF patients. In the echocardiographic findings, morphological abnormalities were observed in both groups, notably, the LAVI exceeded the thresholds for cardiac structure even in the pre‐HFpEF patients. Additionally, the GLS, an indicator of potential left ventricular systolic dysfunction, was significantly reduced in the HFpEF patients.

Both groups exhibited aortic stiffness beyond the normal range; notably, HFpEF patients demonstrated significantly elevated baseline d‐ROMs values compared to pre‐HFpEF patients, suggesting increased oxidative stress.

### 3.2 Effects on Primary Efficacy Endpoints

**FIGURE 2:**
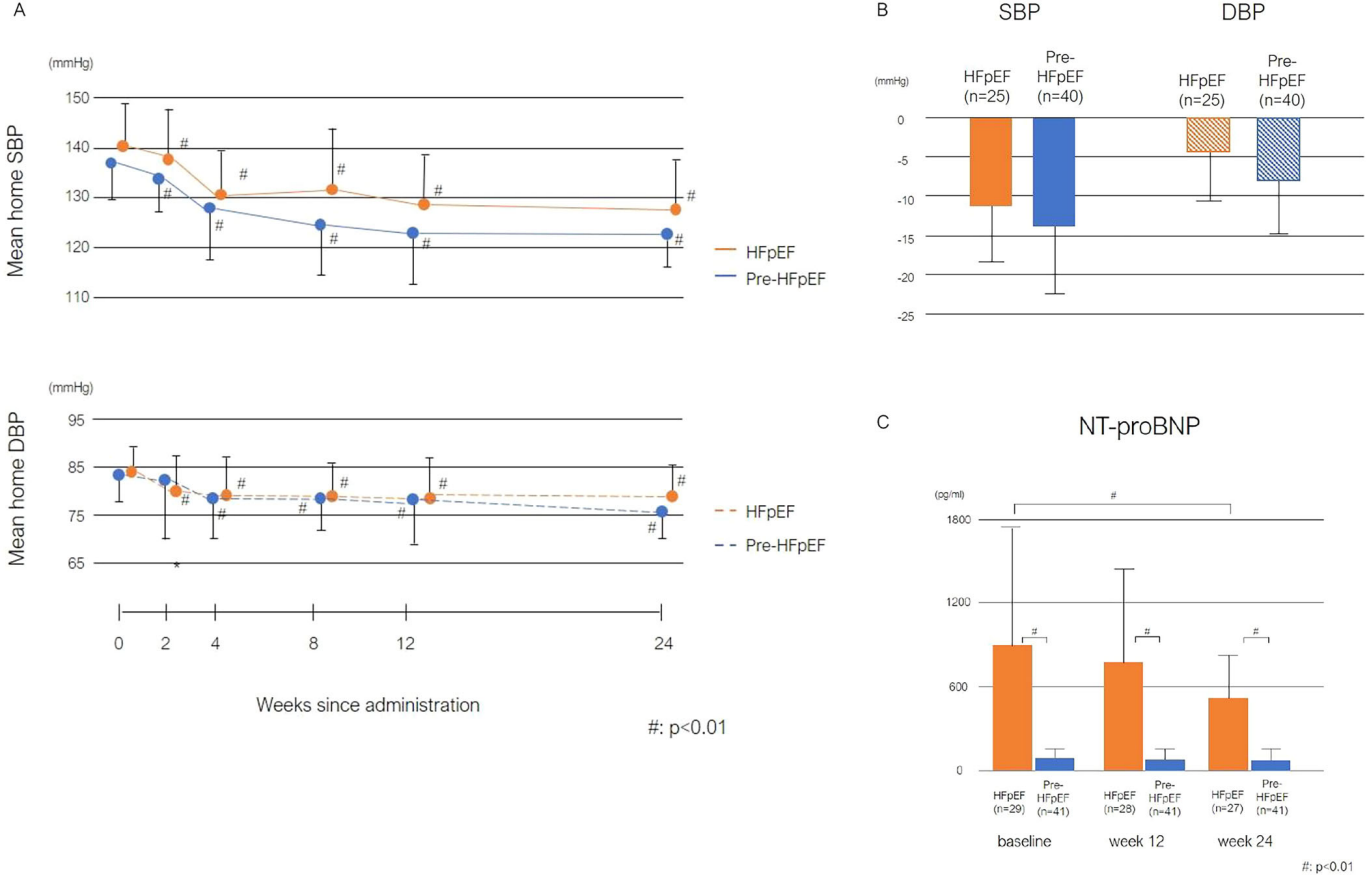
Primary efficacy outcomes in HFpEF and pre‐HFpEF patients over 24 weeks. (A) Changes in home blood pressure over 24 weeks. This graph presents the mean systolic blood pressure (SBP) and diastolic blood pressure (DBP) at home over 24 weeks post‐administration in HFpEF and pre‐HFpEF patients. Significant changes are denoted by # (*p* < 0.01). (B) Comparative reductions in home SBP and DBP. This bar graph illustrates the mean reductions in SBP and DBP from baseline to the study endpoint in HFpEF and pre‐HFpEF patients. SBP indicates systolic blood pressure; and DBP, diastolic blood pressure. (C) NT‐proBNP levels at baseline, week 12, and week 24. This bar chart displays NT‐proBNP levels at baseline, week 12, and week 24 in the both HFpEF and pre‐HFpEF patient. Data are shown as the mean ± standard deviation. Significant differences are indicated by # (*p* < 0.01).

During the 24‐week ESAX treatment period, significant changes in blood pressure were observed in both HFpEF and pre‐HFpEF patients, as illustrated in Figure 2A,B. The daily dosage of ESAX was consistent between the groups at the end of the study (HFpEF vs. pre‐HFpEF = 3.3 ± 1.6 mg vs. 3.2 ± 1.6 mg, *p* = 0.794).

The mean reductions in home SBP/DBP were −11.3 ± 6.9/−4.4 ± 6.2 mmHg in the HFpEF patients, and −13.7 ± 8.8/−8.1 ± 6.9 mmHg in the pre‐HFpEF patients, both showing statistically significant reductions compared to baseline (*p* < 0.001). However, the degree of blood pressure reduction did not significantly differ between the two groups. Notably, a statistically significant decline in NT‐proBNP levels was observed exclusively in the HFpEF group, with a mean reduction of 494.3 ± 304.9 pg/mL at 24 weeks (*p* < 0.001), compared to a decrease of 82.4 ± 79.6 pg/mL in the pre‐HFpEF group (*p* = 0.053; Figure 2C). The percentage decrease in NT‐proBNP levels averaged 26% in the HFpEF patients compared to only 4% in the pre‐HFpEF (*p* = 0.19; intergroup comparison). Another primary endpoint, echocardiographic evaluations, demonstrated significant improvements in both groups, with the exception of the e′ wave velocity. The improvements in the E/e′ ratio and GLS were significantly greater in the pre‐HFpEF patients, as shown in Table 2. By the end of the study, 12 pre‐HFpEF patients (30%) met the criteria for stage A, characterized by NYHA class I, NT‐proBNP levels below 125 pg/ml, and values below established thresholds for cardiac structure and functional abnormalities. No significant changes were observed in BMI and HbA1c levels during the study across both groups.

**TABLE 2:** Echocardiographic parameters and their improvements after ESAX treatment in HFpEF and pre‐HFpEF patients.

	Final value HFpEF pre‐HFpEF	Mean improving rate (%) HFpEF pre‐HFpEF	*p* value comparing Baseline and after 24 weeks	*p* value for inter‐groups
LAVI (mL/m^2^)	37.4 ± 14.1	18.7 ± 16.7	< 0.001	0.104
	31.0 ± 6.3	12.8 ± 9.5	< 0.001	
LVMI (g/m^2^)	106.2 ± 37.3	6.3 ± 10.3	< 0.001	0.645
	84.2 ± 19.5	7.6 ± 12.4	< 0.001	
Septal e′ wave velocity (cm/s)	5.4 ± 1.1	6.8 ± 15.9	0.080	0.643
	7.5 ± 1.6	7.1 ± 13.8	0.051	
E/e′ ratio	12.8 ± 2.7	5.0 ± 16.1	0.026	0.009
	9.0 ± 2.2	16.8 ± 20.2	< 0.001	
GLS (%)	15.1 ± 2.3	5.4 ± 11.2	0.025	0.004
	18.8 ± 4.1	18.5 ± 21.8	< 0.001	

*Note:* Data are mean ± standard deviation.

Abbreviations: GLS, global longitudinal strain; LAVI, left atrial volume index; LVMI, left ventricular mass index.

### 3.3 Effects on Secondary Efficacy Endpoints

**FIGURE 3:**
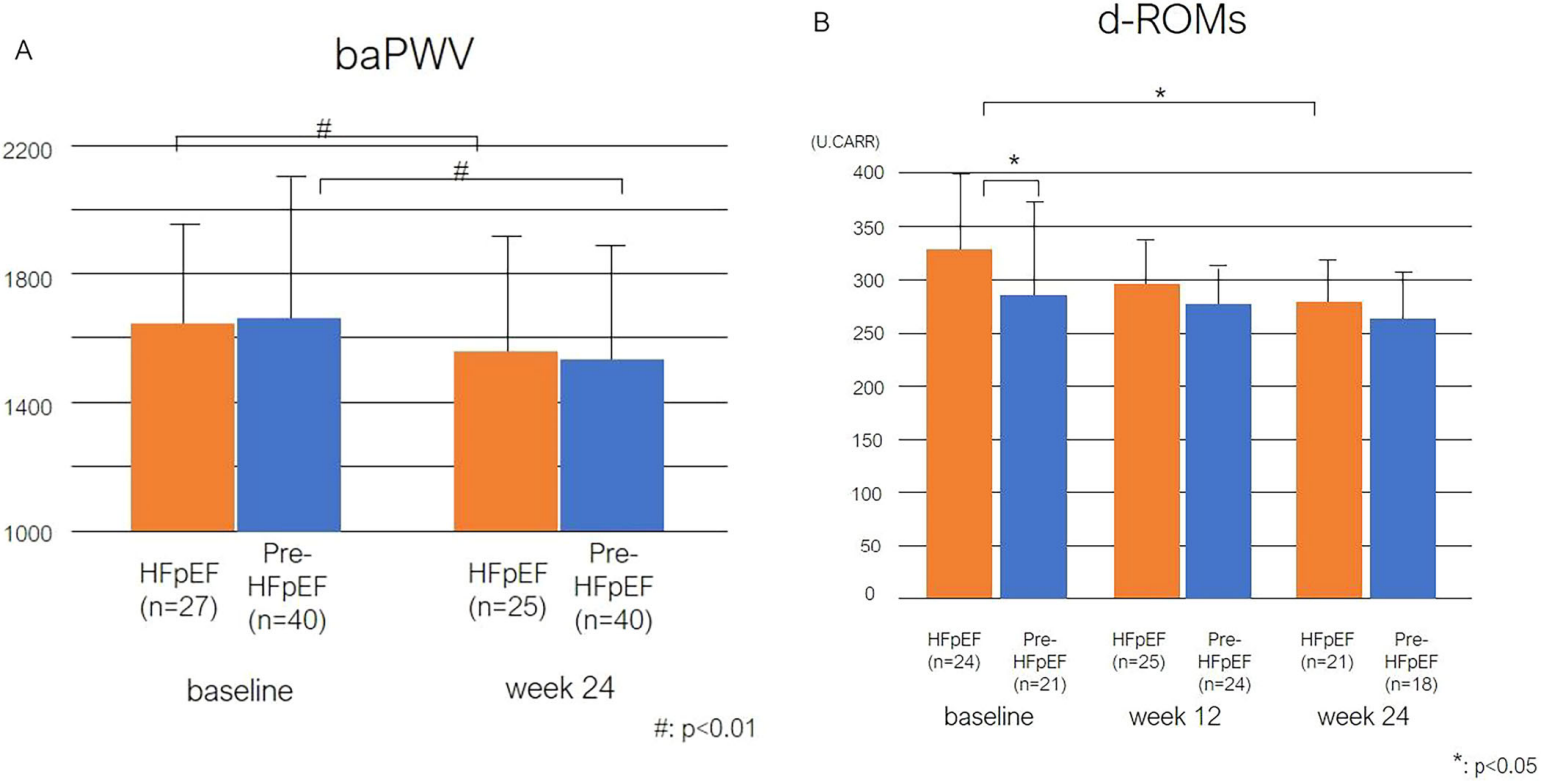
Secondary efficacy endpoints of the study. (A) Changes in bilateral ankle‐brachial pulse wave velocity (baPWV). This bar graph presents the baPWV values at baseline and at week 24 in both HFpEF and pre‐HFpEF patient. Data are shown as the mean ± standard deviation. Significant differences are indicated by # (*p* < 0.01). (B) Reactive oxygen metabolites (d‐ROMs) levels. This bar graph shows the levels of d‐ROMs at baseline, week 12, and week 24 in both HFpEF and pre‐HFpEF patient. Data are shown as the mean ± standard deviation. Statistical significance is indicated by * (*p* < 0.05).

An improvement in aortic stiffness was significantly observed in both groups (Figure 3A). Similarly, d‐ROMs levels also demonstrated significant improvement only in the HFpEF group, with no significant differences observed between the two groups at the end of the observation (HFpEF, 285.7 ± 40.6 U.CARR at 24 weeks; *p* ≤ 0.001 and pre‐HFpEF, 272.5 ± 34.8 U.CARR at 24 weeks; *p* = 0.364 compared to baseline, respectively, Figure 3B).

### 3.4 Safety Aspects

The incidence of all drug‐related adverse events was approximately 40%, with no significant differences between the two groups (Table S1.). Hyperkalemia, a key concern, was observed in four HFpEF patients and two pre‐HFpEF patients; however, no cases of severe hyperkalemia were observed during the treatment period. Only two HFpEF patients were withdrawn from the study due to WRF.

## 4 Discussion

ESAX, a novel, selective nonsteroidal MRA, demonstrates exceptional affinity for MR, showcasing over 1000 times more selectivity compared to glucocorticoid, progesterone, or androgen receptors. This high specificity is critical in effectively managing hypertension, offering an advantageous profile for minimizing common side effects such as hyperkalemia often associated with conventional MRAs such as spironolactone or eplerenone [9, 10].

The modulation of the RAAS particularly through MR involvement, has historically been pivotal in managing heart failure. Recent findings indicate that RAAS is also overly activated in HTN, obesity, and diabetes mellitus, which are early stage in the continuum of heart failure and represent key comorbidities and pathophysiological substrates of HFpEF patients [1, 2, 3, 4, 5, 6]. Accordingly, this study aims to further elucidate the therapeutic benefits and safety profile of ESAX, emphasizing its unique role in the management of HFpEF patients.

Despite the lack of consensus on HFpEF or pre‐HFpEF diagnosis, with various guidelines and reports describing it as a condition in patients showing cardiac structural abnormalities or damage, with or without heart failure symptoms [18]. The present study demonstrated that, although individuals with multiple risk factors for heart failure were recruited, most had already progressed to either a precursor stage or fulfilled the established criteria for HFpEF, consistent with previously reported guidelines, regardless of their condition's severity.

Moreover, our study demonstrated the clinical benefit of ESAX and supports its potential efficacy in these patient groups.

Overall, ESAX treatment demonstrated a significant reduction in blood pressure consistent with previous reports as well as a significant improvement in aortic stiffness.

In HFpEF patients, our findings suggest that ESAX may improve aortic stiffness and lower blood pressure via reduction of oxidative stress. Similarly, our results imply that ESAX may lower NT‐proBNP levels and enhance cardiac function. The existing literatures indicate that MR overactivation contributes to increased oxidative stress and arterial stiffness, exacerbating cardiovascular complications in HFpEF patients [23, 24]. Our study suggests that by regulating MR overactivation, ESAX not only mitigates oxidative stress but also enhances vascular function, thus providing cardiovascular protection. While reductions in oxidative stress have been documented with spironolactone [25], the application of ESAX in achieving comparable outcomes in HFpEF has been less thoroughly investigated. These preliminary findings underscore the potential utility of ESAX as a therapeutic option for managing HFpEF.

The therapeutic benefits of ESAX were particularly evident in pre‐HFpEF patients. These patients exhibited significant improvements in E/e′ ratio, indicative of diastolic function, and in GLS, suggesting potential systolic dysfunction compared to those with HFpEF. Although these changes seem to be blood pressure‐dependent, it is noteworthy that these effects were observed despite uniform blood pressure reductions across all patients. Previous research has demonstrated that RAAS inhibitors, including MRAs, improve cardiac dysfunction in HFpEF patients [26, 27]. However, evidence of such benefits from ESAX remains limited thus far.

Furthermore, in the present study, 30% of pre‐HFpEF patients successfully reverted to a more preliminary stage within the heart failure continuum by the end of the trial, suggesting that early intervention with ESAX aligns with the primary aims of heart failure management, which focus on mitigating progression and preventing new‐onset heart failure. The TOPCAT study of spironolactone in HFpEF patients did not significantly reduce cardiovascular mortality, yet it evidenced a notable reduction in hospitalizations for heart failure, highlighting its potential therapeutic benefits in managing HFpEF [8]. In these subjects, patients with high NT‐proBNP levels associated worse diastolic dysfunction, and spironolactone's ability to reduce NT‐proBNP levels has been associated with positive primary outcomes, consistent with our findings [28, 29]. While spironolactone has been effective in treating hypertension, its primary use has been in clinical scenarios characterized by overactivated RAAS, such as resistant hypertension and primary aldosteronism, with few studies demonstrating its efficacy in the early stages of the heart failure continuum [15, 30, 31]. Recent studies using Finerenone, a nonsteroidal and selective MRA similar to ESAX, support our perspectives. The analysis demonstrated that Finerenone significantly reduced the incidence of cardiovascular outcomes, including new‐onset heart failure in patients with DM and CKD [32]. These findings underscore its efficacy in preventing heart failure and suggest a broader role for nonsteroidal MRAs in heart failure management.

The safety of ESAX, as demonstrated in previous reports, was confirmed in the present study. Previously, hyperkalemia has been a concern with conventional MRAs, limiting their use [33, 34]. In our study, approximately 10% of patients exhibited hyperkalemia, but none required treatment discontinuation. Those results can be attributed to the use of loop diuretics in 30% of the study subjects and the potential involvement of SGLT2 inhibitors. The EMPEROR‐Pooled study, which utilized empagliflozin in heart failure patients, reported a reduced incidence of hyperkalemia [35]. Our study noted WRF in two instances, resonating with the SPRINT study's findings on intensified blood pressure control leading to significant eGFR decline. This suggests that the observed WRF could be attributed to blood pressure reduction.

Our evidence supports ESAX as a viable therapeutic option for HFpEF and pre‐HFpEF patients. Early ESAX intervention in at‐risk patients may curb HFpEF progression, potentially establishing a new standard in management. Nonetheless, further extensive and detailed observational studies are warranted to explore unresolved areas. It is crucial to acknowledge the limitations of our single‐center observational study. These include potential biases and limited generalizability due to the small sample size. Moreover, the study did not have a control arm, and the treatment with ESAX was additive to existing RAAS inhibitor therapy. However, specific dosages and administration methods for these inhibitors were not detailed.

## 5 Conclusion

Our findings strongly support the use of ESAX in the management of heart failure, highlighting its benefits in reducing blood pressure and aortic stiffness in both HFpEF and pre‐HFpEF patients thereby addressing key elements of the disease's pathology. Furthermore, in patients with HFpEF, ESAX reduced NT‐proBNP levels and oxidative stress; in patients with pre‐HFpEF, ESAX more significantly improved cardiac dysfunction, the pathological basis of HFpEF. The safety profile of ESAX was consistent with previous reports. This study may highlight that ESAX treatment can be a fundamental treatment approach in the HFpEF continuum and the importance of early intervention with this therapy. Future research should aim to further validate these findings and explore the long‐term outcomes and safety of ESAX treatment in a larger, more diverse patient population.

## Conflicts of Interest

The authors declare no conflicts of interest.

## Supporting information

Supplementary Table 1.

## Data Availability

The data that support the findings of this study are available on request from the corresponding author. The data are not publicly available due to privacy or ethical restrictions.
