# Supplementary material for: The Role of Esaxerenone in the Continuum of Heart Failure With Preserved Ejection Fraction: Insights From a Prospective Observational Study
Source: Clin Cardiol. 2025 Apr 24;48(4):e70137. doi: 10.1002/clc.70137 (PMC12019700; doi:10.1002/clc.70137)
Supplement: Supplementary file 1 — Supplementary Table 1. [file CLC-48-e70137-s001.docx]

Supplementary Table 1.

|  | **Total**  **(n=67)** | **HFpEF**  **(n=27)** | **Pre-HFpEF (n=40)** | **p value** |
| --- | --- | --- | --- | --- |
| **All drug related adverse events, %** | 25 (37) | 13 (48) | 12 (30) | 0.081 |
| **Drug reaction, n (%)** | 17 (25) | 7 (26) | 10 (25) | 1.000 |
| Dizziness, n (%) | 3 (4) | 1 (4) | 2 (5) | 1.000 |
| Fainting, n (%) | 14 (21) | 6 (22) | 8 (20) | 1.000 |
| **Abnormalities on laboratory test, n (%)** | 8 (12) | 6 (22) | 2 (5) | 0.058 |
| WRF, n (%) | 2 (3) | 2 (7) | 0 (0) | 0.464 |
| Hyperkalemia at any visit, n (%) | 6 (9) | 4 (15) | 2 (5) | 0.402 |
| Severe Hyperkalemia, n (%) | 0 (0) | 0 (0) | 0 (0) | 1.000 |

Supplementary Table 1. Treatment-emergent adverse events

WRF indicates worsening renal function.
